# Supplementary material for: Simplifying principles underlying the highly complex peptide motif of the promiscuous chicken MHC class I molecule, BF2*21:01
Source: eLife. 2026 Nov 2;15:RP111098. doi: 10.7554/eLife.111098 (PMC13727077; doi:10.7554/eLife.111098)
Supplement: Peer review history

# Editor report: eLife Assessment

**DOI:** 10.7554/eLife.111098.3.sa0

**Authors:** Jungsan Sohn

**Affiliations:** Johns Hopkins University School of Medicine United States

**Keywords:** Compelling, Important

This **important** study investigates the peptide-binding principles by promiscuous chicken MHC molecules. The data from crystallography, mass-spec, and modelling are **compelling**. This paper will be of broad interest to immunologists and those interested in vaccine development.

# Referee report: Reviewer #1 (Public review)

**DOI:** 10.7554/eLife.111098.3.sa1

[Editors' note: this version has been assessed by the Reviewing Editor without further input from the original reviewers. The authors have addressed the comments raised in the previous round of review.]

Summary:

Combining in vitro refolding, SEC-based assembly assays, peptide-library screening, MALDI-TOF, LC-MS/MS, structural analysis and immunopeptidomics, this manuscript investigates the peptide-binding principles of the promiscuous chicken MHC-I molecule BF2*21:01.

Strengths:

Although the peptide motif of BF2*21:01 is highly complex, this manuscript identified several principles, including a preference for 10-mer peptides, co-variation between P2 and Pc-2, effects of P3 and Pc-3, and a strong cellular preference for Leu at Pc. The results are important for avian MHC biology and poultry vaccine epitope prediction.

# Referee report: Reviewer #2 (Public review)

**DOI:** 10.7554/eLife.111098.3.sa2

Summary:

The study presents an in-depth analysis of the peptide repertoire bound by a promiscuous chicken MHC molecule using mass spectrometry, x-ray crystallography and modelling. While the MHC can bind a very diverse set of peptides, the authors have found some new rules that govern peptide binding to this MHC that could help to build a predictive model to study the repertoire of pathogen-derived peptides.

Strengths:

The study uses a range of well performed experiment across multiple techniques and provides an in-depth analysis of the peptide repertoire, including peptide sequences, length, preferred residues, stability and MHC presentation.

# Author comment: Author response

**DOI:** 10.7554/eLife.111098.3.sa3

**Authors:** Michael Harrison, Paul E Chappell, Samer Halabi, Maria Danysz, Enock M Mararo, Lukasz Magiera, Clemens Hermann, Michael J Deery, Kathryn S Lilley, Hans-Joachim Wallny, David W Avila, William Mwangi, Venugopal Nair, Susan M Lea, Nicola Ternette, Jim Kaufman

**Affiliations:** University of Cambridge Cambridge United Kingdom; University of Oxford Oxford United Kingdom; University of Edinburgh Edinburgh United Kingdom; University of Edinburgh Edinburgh United Kingdom; University of Edinburgh Edinburgh United Kingdom; University of Cambridge Cambridge United Kingdom; University of Cape Town Cape Town South Africa; University of Cambridge Cambridge United Kingdom; Univerity of Cambridge Cambridge United Kingdom; Basel Institute for Immunology Basel Switzerland; Basel Institute for Immunology Basel Switzerland; The Pirbright Institute Compton United Kingdom; Pirbright Institute Compton United Kingdom; University of Oxford Oxford United Kingdom; University of Oxford Oxford United Kingdom; Yale University New Haven United States

The following is the authors’ response to the original reviews.

**eLife Assessment**

This important study investigates the peptide-binding principles of promiscuous chicken MHC molecules. The data from crystallography, mass spectrometry, and modeling are convincing. However, the presentation would benefit from streamlining and clear links between data and conclusions. This paper will be of broad interest to immunologists and those interested in vaccine development.

Overall, we are delighted and grateful to the eLIFE editors and the two reviewers for the careful and thoughtful assessments and reviews of our paper. We are glad that the strengths of the paper were apparent and appreciated. And of course, every paper has weaknesses, especially for a story as complex as this one.

We made only minor changes to accommodate the reviewer comments, along with additions for which we only became aware upon this submission of a revised manuscript. In particular, we shortened the title and abstract to fit what is usual for an eLIFE paper, added Key Resources table with accompanying references, changed the numbering of the figures throughout the manuscript to ensure that each page represented a figure (rather than panels of a figure), moved the figure legends from the embedded figures to a list near the end of the manuscript, and split the supplemental spreadsheet into two renamed Data Source files.

Before answering the comments and questions directly, perhaps a few points would help clarify why the paper is as it is.

First, the experiments cover over three decades of work, with the first gas phase sequencing results done in 1992. Unlike some of the chicken class I alleles which immediately gave completely clear stringent motifs (B4, B12 and B15 in Wallny et al 2006 PNAS, B19 in Han et al 2023 J Immunol), we harvested nothing but confusion from the B21 class I results (Fig. 1). Initially, we thought that the lack of a clear motif for B21 was due to multiple well-expressed class I molecules but only one dominantly-expressed class I molecule was found (Wallny et al 2006 PNAS, Shaw et al 2007 J Immunol) and, to our surprise, bacterially-expressed BF2*21:01 heavy chain and b_2_-microglobulin refolded with two synthetic peptides without sequence in common, and the crystal structures showed that this molecule remodeled the binding site to accommodate two such disparate peptides (Koch et al 2008 Immunity). This was the beginning of our understanding of the spectrum of class I alleles from promiscuous generalists to fastidious specialists, which we have explored in a series of further papers (in particular, Chappell et al 2015 eLIFE, Tresgaskes et al 2016 PNAS, Kaufman 2018 Trends Immunol, Tregaskes and Kaufman 2022 Mol Immunol).

Second, over these many years, we continued to explore the binding properties of BF2*21:01 in ever more detail, resulting in the current manuscript. We learned only slowly how to probe this unexpected promiscuity, unprecedented in the MHC literature, so that the experiments proceeded with our best understanding at the time, including taking advantage of new approaches as they become available. Each experiment built on the previous set of experiments and each brought us closer to an understanding.

Third, having amassed a collection of data, we chose eLIFE exactly because it allows us to present the entire story from beginning to end without compromise, not just the highlights with the major points illustrated by a few main figures and with the supporting data in many supplementary figures. We include all the data, because it is all part of the story, and so interested researchers to look at the data from their own perspective. Although mostly we provide bar graphs, we include spreadsheets for the raw data (or close to them) for the final experiments (illustrated by Figs. 10 and 14-22) in the two source data files, so these can be assessed easily by others in the field, perhaps using approaches that we may not feel competent to perform.

**Public Reviews:**

**Reviewer #1 (Public review):**

Summary:

Combining in vitro refolding, SEC-based assembly assays, peptide-library screening, MALDI-TOF, LC-MS/MS, structural analysis and immunopeptidomics, this manuscript investigates the peptide-binding principles of the promiscuous chicken MHC-I molecule BF2*21:01.

Strengths:

Although the peptide motif of BF2*21:01 is highly complex, this manuscript identified several principles, including a preference for 10-mer peptides, co-variation between P2 and Pc-2, effects of P3 and Pc-3, and a strong cellular preference for Leu at Pc. The results are important for avian MHC biology and poultry vaccine epitope prediction.

Weaknesses:

The manuscript is sometimes difficult to follow because the authors present a large amount of peptide-library, structural and immunopeptidomics data. without always clearly explaining how these datasets support the proposed simplifying principles.

We are delighted and grateful to the reviewer 1 for the careful and thoughtful comments and questions concerning our manuscript. We are glad that the strengths of the paper were apparent and appreciated, and acknowledge the weaknesses that come with such a complex story with experiments performed over decades.

Major Issues - Points Requiring Clarification or Additional Support:

(1) Line 282-301, 537-545）

The immunopeptidomics conclusions are mainly based on one B21 cell line with one biological replicate and at least two technical replicates. Given the complexity of the BF2*21:01 peptide repertoire, this is a major limitation. The authors should either provide additional biological replicates or clearly state this limitation in the Abstract, Results and Discussion.

This limitation is clearly stated in lines 537-545, as part of a paragraph covering the various ways in which the data presented in this manuscript could be improved. In fact, we have performed immunopeptidomics of several different B21 cell types, with many replicates and found similar data as presented, giving us confidence in our interpretations. However, these other experiments belong in different stories, so it is not appropriate that the data be reported in this manuscript.

(2) (Lines 290-313)

The B21 cell preparations contain both BF2 and the lowly expressed BF1 molecule. Some peptides, especially 8-mers or peptides with atypical motifs, may derive from BF1*21:01. The authors should clarify how BF2*21:01-bound peptides were distinguished from possible BF1-derived peptides, or interpret the immunopeptidomics motif more cautiously. The authors should also provide or cite evidence confirming the B21 haplotype identity of the cell line and chicken materials used for immunopeptidomics.

The concern about the contribution of BF1*21:01 to the immunopeptidomics is clearly stated in the manuscript, both lines 290-313 and as part of the paragraph describing the limitations of the experiments (lines 542-543). In fact, the expression of BF1 molecules has long been known to be less than 10% of BF2 molecules at the RNA level, and much less at the protein level (Wallny et al 2006 PNAS, Shaw et al 2007 J Immunol). The proportion of 8mers identified by immunopeptidomics is also low (Fig. 14), and it is not impossible that most 8mers are due to BF1*21:01. We have used assembly assays with peptide libraries, immunopeptidomics and a crystal structure to determine the peptide motif for typical BF1 molecules, of which BF1*21:01 is one and found it may contribute to 8mer peptides but very seldom to longer peptides. This work is unpublished but gives us confidence that the characteristics of BF2*21:01 are not misrepresented by the data in this manuscript.

The sources of the chicken samples and the cell lines are described in detail under Materials and Methods (lines 577-590), citing relevant publications.

(3) (Lines 217-221, 243-253)

The authors acknowledge that MALDI-TOF cannot reliably distinguish peptide combinations with identical or similar masses, nor determine residue positions in some cases. Therefore, MALDI-TOF results should not be overinterpreted as precise evidence for residue preference. The authors should clearly indicate which conclusions are supported by LC-MS/MS.

As described, the experiments follow each other in temporal sequence, so that we started with single peptides, then peptide libraries that varied in one position, then peptide libraries that varied in two positions first analysed by MALDI-TOF and later by LC-MS/MS. The final experiment (Fig. 10, with the original data in the supplementary spreadsheet) directly compares MALDI-TOF and LC-MS/MS results for six peptide libraries, so that the strength of the evidence for residue preference is clear. Throughout the manuscript, we do our best to not to overstate conclusions based on the data of any particular experiment.

(4) (Lines 297-301, 316-330)

The authors suggest that longer peptides may bulge in the middle or extend out of the groove at the C-terminal end. The rationale for the C-terminal extension is not clearly explained. Why is the C-terminal extension considered rather than the N-terminal extension? If the binding register is uncertain, long peptides should be analyzed separately from canonical-length peptides.

When the first sequence of a chicken class I cDNA was determined, an immediate mystery was why one of the so-called invariant residues that coordinate the N- and C-termini of the bound peptide is not conserved (Kaufman et al 1992 J Immunol). In fact, this residue Tyr at position 86 in HLA-A2 and the equivalent position in all mammalian classical class I molecules is an Arg in the classical class I molecules of all non-mammalian vertebrates and is common with class II molecules (Kaufman et al 1995 Semin Immunol). Similar to class II molecules, this Arg in chicken class I molecules allows the peptide to extend out of the C-terminus, as shown by a crystal structure (Xiao et al 2018 J Immunol). The concern that we might be misidentifying the C-terminal amino acid was the basis for the analysis in Figs. 23 and 24, but in the absence of crystal structures, we are not able to provide a final answer this question. Perhaps relevant is the fact that a chicken class II molecule can bind exactly the same peptide in two conformations, one with a canonical 9mer core and the other with an unexpected 10mer core (Goryanin et al 2026 J Virol).

By contrast, N-terminal extensions are only found for some class I alleles and thus far depend on the substitution of small amino acid sidechains for W166 (Li et al 2011 J Virol for bovine, Ma et al 2020 J Immunol for Xenopus, Wei et al 2022 J Immunol for ovine). Thus far, no chicken BF2 sequences have this substitution, consonant with the many crystal structures, including those for BF2*21:01 (Koch et al 2008 Immunity, Chappell et al 2015 eLIFE, this manuscript). However, in unpublished data, we find that most BF1 sequences have sequence differences that could allow N-terminal extensions, although we have no crystal structures to support this possibility.

(5) (Lines 406-439)

In vitro assembly assays show that several hydrophobic residues can be tolerated at Pc, whereas immunopeptidomics shows a strong Leu preference at this position. The authors should clarify whether this Leu preference reflects intrinsic BF2*21:01 binding specificity, TAP-mediated peptide transport, antigen processing, peptide loading, or a cell-line-specific effect. Additional experimental support, such as TAP transport analysis, would strengthen this conclusion.

The preference for Leu at the final position of the peptide by immunopeptidomics of the B21 cell line is strong but not absolute and is certainly affected at the least by the length of the peptide (Figs. 23 and 24). Unpublished immunopeptidomics results (mentioned above) show that this is not a cell line-specific result. The evidence from assembly assays of various peptides is that several hydrophobic amino acids are tolerated with sufficient stability of BF2*21:01 that they are detected in the assay (Figs. 3, 5, 9 and 10). Thermostability assays (Fig. 6) show that peptides with these same hydrophobic amino acids are stable to at least body temperature of chickens. These experiments show that such stability is peptide-dependent (that is, whether a particular amino acid is tolerated depends on the stability conferred by the rest of the peptide). Finally, peptide translocation assays using B21 cells have been done (Tregaskes et al 2016 PNAS) and show that peptides with several hydrophobic amino acids can be pumped into the lumen of the endoplasmic reticulum. However, the assays are with single synthetic peptides, so the data are not extensive enough to separate the effects of the final amino acid from the rest of the peptide. Certainly, peptides with amino acids other than Leu at the C-terminus can be translocated. So, it is not yet clear at which point the preference for Leu at the C-terminus of the peptide arises.

(6) (Lines 172-178, 243-279, 442-457)

The structural analysis explains some residue combinations, such as Arg at P2 with Glu at Pc-2 or Trp at Pc. However, the structural interpretation is not fully integrated with the large-scale peptide library and immunopeptidomics results. Representative high- and low-frequency combinations should be discussed structurally.

Six crystal structures show that BF2*21:02 remodels the binding to accommodate a variety of anchor residues (Koch et al 2008 Immunity, Chappel et al 2015 eLIFE). These crystal structures are representative of sequences found by the immunopeptidomics from very frequent (H-E at roughly 15% 8-12mers) to moderately frequent (E-L at roughly 6% 8-12mers) to infrequent (N-F, A-D and E-D at roughly 1.5%, 1.6% and 0.7% 8-12mers) based on Fig. 18. All but one of the structures has Leu at the C-terminus, with the last one having Val which is found but not frequently by immunopeptidomics.

Similar numbers are found by LC-MS/MS of double-substitution libraries of the two original peptide sequences in Fig. 10 with H-E found frequently (8.1% in P390, 3.8% in P498) and the others infrequently (0.1, 0.9, 1.0, 0.3% in P390, 0, 1.4, 1.0, 0.3% in P498), as calculated from the numbers in the Supplementary data spreadsheet. As discussed in the manuscript, for single-substitution peptide libraries of the two original peptides, Ile/Leu at the C-terminus was very frequent but at the same or slightly less level as Phe, with Met less frequent and Val even less so (Fig. 7).

In addition, there are two more structures along with models explicitly testing some substitutions (Fig. 5). Attempting more current modelling approaches, we found AlphaFold 3 was unable to correctly predict most of the conformations that are found in the crystal structures of BF2*21:01, so we don’t feel confident in using them to predict unknown structures of this kind.

(7) The inference of co-variation between P2 and Pc-2, as well as the modulatory effects of P3 and Pc-3, should be better explained. At present, some conclusions appear to be based mainly on residue-frequency patterns, and the logical connection between these observations and the proposed binding principles is not always clear. Statistical analyses, such as mutual information, chi-square tests or permutation tests, and representative structural explanations would strengthen this conclusion.

We endeavored to do our best to explain the data, our interpretations and our reasoning, so we apologise if we have not managed to be as clear as might be desired. We have included as close to raw data as possible for the LC-MS/MS and MALDI-TOF (Fig. 10) and for the immunopeptidomics (Fig. 14 and 18) in the Supplementary Data spreadsheet, exactly so that competent practitioners can carry out further analyses (including the sophisticated statistical tests mentioned).

**Reviewer #2 (Public review):**

Summary:

The study presents an in-depth analysis of the peptide repertoire bound by a promiscuous chicken MHC molecule using mass spectrometry, x-ray crystallography and modelling. While the MHC can bind a very diverse set of peptides, the authors have found some new rules that govern peptide binding to this MHC that could help to build a predictive model to study the repertoire of pathogen-derived peptides.

Strengths:

The study uses a range of well performed experiment across multiple techniques and provides an in-depth analysis of the peptide repertoire, including peptide sequences, length, preferred residues, stability and MHC presentation.

Weaknesses:

The data overall support the analysis and conclusion well. The only caveat is linked to Figure 4, which does not describe the stability of the peptide-MHC complex, but instead shows refold yield, and the two are not always linked.

We are grateful for the clear understanding of the strengths of the work. With regards to Fig. 4, we agree with the reviewer that there are differences in refold yield but that measure may not be correlated with stability of the peptide-MHC complex. However, we were basing our interpretation of stability on the position and quality of the monomer peak, as illustrated by the trace in Fig. 2, in which a sharp peak at the monomer position represents a stable complex (as seen for the 10 and 11mer peptides) and later peaks represent unstable complexes falling apart during the chromatography (as seen for the 7, 8 and 9mer peptides).

**Recommendations for the authors:**

**Reviewer #1 (Recommendations for the authors):**

Minor Issues: Editorial and Data Presentation Modifications

(1) Lines 53-62, 155-170, 303-314: The terms Pc, Pc-2 and Pc-3 should be clearly defined early in the manuscript and figure legends.

The Abstract introduces the abbreviations as “peptide positions P_2_ and P_c-2_” followed by P_C_, which are standard usage and seem clear. The first usage in the text is “anchor residues at three positions, with co-variation between the anchor residues at P_2_ and P_c-2_” which again seems clear, particularly in the context of the text and the figure. However, a parenthetical description has been added to read “…anchor residues at three positions, with co-variation between the anchor residues at P_2_ and P_c-2_ (position 2 and the position two before the C-terminus) …”. Given these usages, it seems unlikely that the reader will fail to understand P_C_ and P_C-3_.

(2) Lines 255-279: The term "peptide backbone" should be defined as the fixed sequence context outside the randomized positions, if this is what the authors mean. Suggest clarifying the meaning of "peptide backbone".

The phrase reads “double-substitution libraries based on four peptide backbones”, which in context of the figures seems clear. However, a parenthetical description has been added: “The examination of double-substitution libraries analysed by MALDI-TOF was expanded to other backbones (that is, other sequences in which two positions were randomised): …”.

(3) Several figures are complex. The authors should add brief take-home messages to figure legends.

Every figure legend starts with a (sometimes quite long) take-home message. It is not clear what more should be added.

(4) A concise summary table of the proposed binding rules, including preferred peptide length, P2, Pc-2 and Pc preferences, and the effects of P3/Pc-3, would be useful for readers.

A concise summary table of binding rules would be very helpful, but the rules are complex, both qualitative and quantitative. For example, immunopeptidomics shows that 10mers are preferred, but that fails to capture the quantitation. The co-variation of P_2_ and P_c-2_, which is the strongest and best characterized correlation, nevertheless is complex since the occupancy of P_2_ by different amino acids (presumably independently of P_c-2_) varies considerably. At our current level of understanding, it is hard to imagine a table that is both concise and precise. With time and more data, perhaps code for quantitative prediction might be constructed (dare one suggest machine learning…), which is the hope of presenting all the available data in one place.

(5) The Results section contains a large amount of peptide-library, structural and immunopeptidomics data. The authors should improve the logical flow and add clearer transition sentences to explain how each dataset supports the proposed simplifying principles.

The reviewer is of course correct that any written text can be improved (although each critic may have a different idea about which part should be fixed), but we have done our best with the material and time available. We could respond productively to a more detailed critique.

(6) Some statements in the Abstract and Discussion should be softened, especially those related to in vivo peptide preferences, BF2*21:01 promiscuity and peptide prediction, given the limited biological replication and uncertainty in peptide assignment.

The statements in the both the Abstract and Discussion are very general, reflecting what we believe to be careful interpretations based on the data. We could respond productively to concerns about specific claims.

**Reviewer #2 (Recommendations for the authors):**

Overall, the data presented in this study are interesting; however, it is complex, and some results could be merged and simplified, as well as the figures. The data provide an in-depth analysis, using mass spectrometry, of the interplay between the different positions of the peptide and the residues favourable to bind within the antigen-binding cleft.

(1) From the abstract, the concept of "promiscuous generalists and fastidious specialists" is not explored after or defined within the results.

The Abstract introduces the concept of promiscuous generalists and fastidious specialists to provide the basis for exploring the peptide-binding specificity of the most promiscuous class I known, BF2*21:01. This overall concept is described in enormous detail in several publications cited in the current manuscript, but it is not particularly germane to the analyses.

(2) From the abstract "These simplifying principles may eventually allow predictions of pathogen peptides", I'm not sure how "simplifying" the principles are with the data, if anything, it does show a rather complex interplay between the different residues of the peptide that enable the MHC to bind a large and diverse number of peptides.

Compared to any combination of anchor residues being permitted at equal frequency, there are clear preferences which the experiments identify. Instead of an enormous range of possible peptide lengths, roughly 50% of peptides are 10mers. Of course, the structural reasons behind these results are certainly complex and likely must be understood in detail in order to attempt peptide predictions.

(3) Line 48. "less well-expressed". Less than what? Do you mean the level of expression was lower? And if yes, are there values of fold change for comparison?

The sentence in the Abstract reads “Chicken BF2 alleles … are less well-expressed on the cell surface … while certain human HLA-B alleles … are well-expressed …”. Read as a complete sentence, the meaning is clear. This difference for chicken BF2 alleles has been quantified as reported in several publications cited in the current manuscript, ranging from 3-5 fold for peripheral blood lymphocytes to ten-fold for erythrocytes (Kaufman et al 1995 Immunol Rev, Chappel et al 2015 eLIFE), with similar numbers for a few HLA-B alleles on human peripheral blood lymphocytes and monocytes (Chappell et al 2015 eLIFE).

(4) Line 149. "with individual peptides" which peptides are we referring to here?

This introductory sentence to a paragraph outlines the general method used for the experiments in this section of the Results, “refolding in vitro … with individual peptides.” Which “individual peptides” are described in the following paragraphs, with the next section of the Results using “refolding in vitro … with peptide libraries”.

(5) Line 170. If 9 mers and below are not stable, which is not really quantified or shown with the data on Figure 4, why is refolding material observed for peptides with different lengths of 9 aa and below on Figure 4? A lower yield of refolded material can have a different origin, and there is no association between stability and refold yield. The notion of stability here probably needs to be changed, as it does not apply to the data.

As described in our response to a similar concern above, we are not basing our interpretation of stability on the quantity of refolded material, but on the position and quality of the monomer peak, as described clearly in the legend to Fig. 4: “The original 10mer (REVDEQLLSV) and 11mer (GHAEEYGAETL) peptides refold with BF2*21:01 to give stable monomers as do 11mer and 10mer derivative peptides, but 9mer, 8mer or 7mer peptides give heavy chain only peaks” and “The peptides 11mer GHAEEYAETL (top panel), 10mer REVDEQLLSV (middle panel), 11mer GHAEAAAAETL and 10mer GHAEAAAETL (bottom panel) gave sharp monomer peaks, while the 9mer GHAEAAETL, 8mer GHAEAETL and 7mer GHAEETL gave a delayed broad peak indicative of unstable binding or heavy chain.” This concept is illustrated by the trace in Fig. 2 (discussed in the text at the beginning of this section of the Results), in which a sharp peak at the monomer position represents a stable complex, and later peaks represent unstable complexes falling apart during the chromatography or free heavy chains.

(6) Line 175. As the 3BEV structure had a P2-His, is a comparable structure expected?

This sentence reads “Structures with amino acid substitutions in the 11mer peptide GHAEEYGAETL bind with Asp at P_c-2_ and either His or Arg at P_2_ (5AD0 and 5ADZ), comparable to the original structure (3BEV) (Fig. 5A).” Minor changes in positions and orientations of individual amino acid sidechains are expected and are clear from the crystal structures presented in Fig. 5A, but are overall comparable to the original peptide in 3BEV, which has a His at P_2_ and a Glu at P_c-2_.

(7) Line 175 "modelling the substituted". How was the modelling done?

The sentence reads “Modelling the substituted peptide with Arg at P_2_ and Glu at P_c-2_ shows a steric clash that can explain why this peptide did not refold with BF2*21:01 (Fig. 5A).” The legend to Fig. 5A states that “modelling done as detailed in Materials and Methods”, but apparently that section was omitted. A section has been added now to the Materials and Methods which states “Beginning with known crystal structures, modelling was carried out using PyMol with the protein mutagenesis Wizard, the rotamer toggle, show bumps and show surfaces.”

(8) Line 176 "shows a steric clash" with what? Figure 5 is too small to see, and there is no label on the residue to follow where the steric clash is coming from.

As stated in the legend to Fig. 5, “Structures were determined for GHAEEYGAETL (3BEV), GHAEEYGADTL (5AD0) and GRAEEYGADTL (5ACZ), which all refolded successful to give stable monomers, while GRAEEYGAETL did not (see Fig. 3), all models of which showed steric clashes (one depicted, red arrow).” In the model shown, the clash is between R9 of the BF2*21:01 with the Glu at peptide position 9. Parenthetically, this depiction of the key MHC residues for the co-variation has been used repeatedly, starting with Chappell et al 2015 eLIFE. The picture can be zoomed to make it large enough to see.

(9) Line 247 "many combinations". It is not clear here if combinations are referring to a set of double-substitutions or different peptides?

Each peptide in the library has a different double-substitution, so the meaning is the same either way. The point of Fig. 10 is to compare the identification of peptides from LC-MS/MS (in which each peptide is identified exactly) with the identification of sets of peptides from MALDI-TOF (in which the order of the double substitution is not clear, as well as the exact amino acid in the cases of I/L and Q/K). As the figure shows, the numbers are generally very similar, but this sentence describes the percentage of cases for which only one method or the other identified a peptide.

(10) Lines 252-253. The conclusion of the MS data that the LC-MS/NS is more accurate and sensitive than MALDI-TOF is not very surprising. What was the rationale for using both?

Our examination of the binding properties of BF2*21:01 for peptides and peptide libraries developed over a long time-span, so in the beginning we looked at single peptides with size exclusion chromatography peaks as the measure, then single- and later double-substitution libraries, first by MALDI-TOF and later by LC-MS/MS. Each set of experiments built on the previous work, so that together they tell the story. For example, after we optimized the use of double-substitution libraries, we were worried about the effects of temperature, so we tested that by MALDI-TOF. While repeating the temperature experiment once we optimized the LC-MS/MS approach might have yielded some additional data, there were other questions to answer.

(11) Line 272-273 "support the idea that P3 is an important position within the peptide (Figures 8-10) despite not contacting the MHC molecule" The structure of 3BEV clearly shows interaction between the P3-Ala and the Tyr156 of the MHC. Residues that are fully or partially buried in the MHC cleft almost all contact the MHC molecule. Maybe I've missed something, but I think this statement is inaccurate.

We agree with the reviewer that nearly every peptide residue contacts the MHC molecule (but of course some much more than others). The statement is now changed in the text to read “support the idea that P_3_ is an important position within the peptide (Figures 8-10) despite not being an anchor residue."

(12) Line 294. Figure 14 clearly shows that the number of 8 and 9-mer peptides eluted is at the same level as the 11mer and above, so how does the data fit with the statement that 9mer and shorter peptides are not stable with the MHC?

Fig. 4 shows that 7, 8 and 9mer derivatives of the original 11mer failed to refold to give a single peak of stable monomers, while Fig. 6 shows that the 10mer derivative of the original 11mer was more thermostable than both the 9mer derivative and the original 11mer. The reason why the 9mer yielded so little monomer in Fig. 4 while giving enough to test by thermostability in Fig. 6 is no longer remembered. A key point is that these results are peptide sequence-specific, so it is not impossible to imagine stable binding of an appropriate 8mer (or perhaps even a 7mer). Another uncertainty, mentioned in the Results and Discussion, is that BF1*21:01 molecules bind primarily 8mers, and the contribution of peptides from BF1*21:01 is not known with certainty.

(13) Line 316. What was the rationale for choosing peptides > 12aa to see if there is some overhang or bulge? As even 11-12mer can exhibit such features.

We were just looking for any obvious patterns, but we didn’t find any.

(14) The section starting at line 366 would have benefited from some structure prediction or modelling to illustrate the findings.

We would have been delighted to model peptides, but we have used AlphaFold3 to compare the models to our crystal structures for seven chicken class I alleles (including BF2*21:01) with one or more peptides. The models sometimes fit the experimental data but they often didn’t, often by a wide margin, and with BF2*21:01 the worst (presumably because the system is not trained on MHC molecules which utilise charge transfer). Therefore, we do not feel confident in using any such modelling approaches except in the most defined situations (such as illustrated in Fig. 5).

(15) Typo - Alleles should be italic, and in vitro as well.

Alleles of genes are in italics, but alleles of proteins are not. To write in vitro in italics is customary, which we have corrected.

(16) Figures

(a) Some of the figures could be merged together.

Of course, any presentation can be improved, but which figures we should merge (some already being three pages in length) is not clear. We could productively respond to more detailed suggestions.

(b) Figure 1. I can't see the different colours mentioned in the figure legend

Our apologies if the colours are not clear enough, but they are present only as an aid (as elsewhere in the manuscript), with red D and E, blue H, K and R, green N, Q, S and T, and all other amino acids black.

(c) Figure 12. Is this figure only with 11mer peptides?

Figure 12 shows the percentage of peptides with particular amino acids at P_2_ and P_c-2_ for three 11-mer double-substitution peptide libraries, the sequences of which are written on the graphs and described in the figure legends.

(d) Table 1. The name of the protein should be added to the table.

OK.
